# Comparison of (*R*)-ketamine and lanicemine on depression-like phenotype and abnormal composition of gut microbiota in a social defeat stress model

**DOI:** 10.1038/s41598-017-16060-7

**Published:** 2017-11-16

**Authors:** Youge Qu, Chun Yang, Qian Ren, Min Ma, Chao Dong, Kenji Hashimoto

**Affiliations:** 1 Division of Clinical Neuroscience, Chiba University Center for Forensic Mental Health, Chiba, Japan; 2 Present Address: Department of Anesthesiology, Tongji Hospital, Tongji Medical College, Huazhong University of Science and Technology, Wuhan, 430030 China

## Abstract

Accumulating evidence suggests a key role of the gut–microbiota–brain axis in the antidepressant actions of certain compounds. Ketamine, an *N*-methyl-D-aspartate receptor (NMDAR) antagonist, showed rapid and sustained antidepressant effects in treatment-resistant depressed patients. In contrast, another NMDAR antagonist, lanicemine, did not exhibit antidepressant effects in such patients. (*R*)-ketamine, the (*R*)-enantiomer of ketamine, has rapid-acting and long-lasting antidepressant effects in rodent models of depression. Here we compared the effects of (*R*)-ketamine and lanicemine on depression-like phenotype and the composition of the gut microbiota in susceptible mice after chronic social defeat stress (CSDS). In behavioral tests, (*R*)-ketamine showed antidepressant effects in the susceptible mice, whereas lanicemine did not. The 16S ribosomal RNA gene sequencing of feces demonstrated that (*R*)-ketamine, but not lanicemine, significantly attenuated the altered levels of *Bacteroidales*, *Clostridiales* and *Ruminococcaceae* in the susceptible mice after CSDS. At the genus level, (*R*)-ketamine significantly attenuated the marked increase of *Clostridium* in the susceptible mice. In contrast, the effects of lanicemine were less potent than those of (*R*)-ketamine. This study suggests that the antidepressant effects of (*R*)-ketamine might be partly mediated by the restoration of altered compositions of the gut microbiota in a CSDS model.

## Introduction

Ketamine is an *N*-methyl-D-aspartate receptor (NMDAR) antagonist. Since the first report of its antidepressant effects in depressed patients by Berman *et al*.^1^, a number of clinical studies have replicated ketamine’s antidepressant effects in treatment-resistant unipolar^2,3^ and bipolar^4,5^ depression. Recent meta-analysis demonstrated that ketamine exhibits rapid and sustained antidepressant effects in treatment-resistant depressed patients^6,7^. Interestingly, ketamine demonstrated a rapid reduction of suicidal ideation in treatment-resistant depressed patients^8,9^. In contrast, it is well recognized that ketamine produces acute transient psychotomimetic side effects after single or repeated infusions^1–5,10,11^. Taken together, ketamine is the most prominent antidepressant for treatment-resistant depression, although the psychotomimetic side effects and abuse potential of ketamine should not be ignored^12–19^. Ketamine’s antidepressant actions are accompanied by the stimulation of the α-amino-3-hydroxy-5-methyl-4-isoxazolepropionic acid receptor (AMPAR), the mammalian target of rapamycin (mTOR), brain-derived neurotrophic factor (BDNF)-TrkB signaling, and increased synaptogenesis^12,20–23^. However, the mechanisms of action through which ketamine exerts its rapid-acting and sustained antidepressant effects are not fully understood.

Ketamine (Ki = 0.53 μM for NMDAR) is a racemic mixture comprising equal parts of (*R*)-ketamine and (*S*)-ketamine. (*S*)-ketamine (Ki = 0.30 μM for NMDAR) exhibits an approximately 3–4-fold greater binding affinity for NMDAR than (*R*)-ketamine (Ki = 1.40 μM for NMDAR), which pharmacologically explains the fact that (*S*)-ketamine has an approximately 4-fold greater anesthetic potency and greater undesirable psychotomimetic side effects than (*R*)-ketamine^13–15,24^. We demonstrated that (*R*)-ketamine has more potent and longer lasting antidepressant effects than (*S*)-ketamine in the animal models of depression^25–29^. Unlike (*S*)-ketamine, (*R*)-ketamine appears to lack psychotomimetic side effects and abuse potential^30,31^.

Lanicemine (AZD6765: (1 *S*)-1-phenyl-2-pyridin-2-ylethaneamine) (Ki = 0.56–1.5 μM for NMDAR) is a low-trapping NMDAR channel blocker sharing many pharmacological effects of ketamine at the NMDAR^32,33^. A single intravenous dose of lanicemine showed rapid but short-lived antidepressant effects in treatment-resistant depressed patients (n = 22)^32^. A subsequent phase IIb study reported that lanicemine (100 or 150 mg, three intravenous infusions per week for 3 weeks, as an adjunct to one antidepressant) was associated with a significant improvement of depressive symptoms in treatment-resistant depressed patients (n = 152)^33^. However, a recent phase IIb study using a larger sample size reported that lanicemine (50 or 100 mg, three intravenous infusions per week for 12 weeks, as an adjunct to one ongoing antidepressant) did not improve depressive symptoms in treatment-resistant depressed patients (n = 302)^34^. These data suggest no evidence to support the efficacy of lanicemine augmentation over placebo in treating treatment-resistant depressive symptoms. Collectively, it is unclear how NMDAR inhibition plays a role in the antidepressant properties of ketamine^14,15^.

Accumulating evidence suggests that the gut microbiota might play a role in the pathogenesis of depression and the antidepressant actions of antidepressants^35–44^. The present study was therefore conducted to examine whether the gut microbiota plays a role in the mechanisms underlying the antidepressant actions of (*R*)-ketamine and lanicemine in a chronic social defeat stress (CSDS) model.

## Results

### (R)-ketamine, but not lanicemine, shows rapid and sustained antidepressant effects in susceptible mice after CSDS

**Figure 1:**
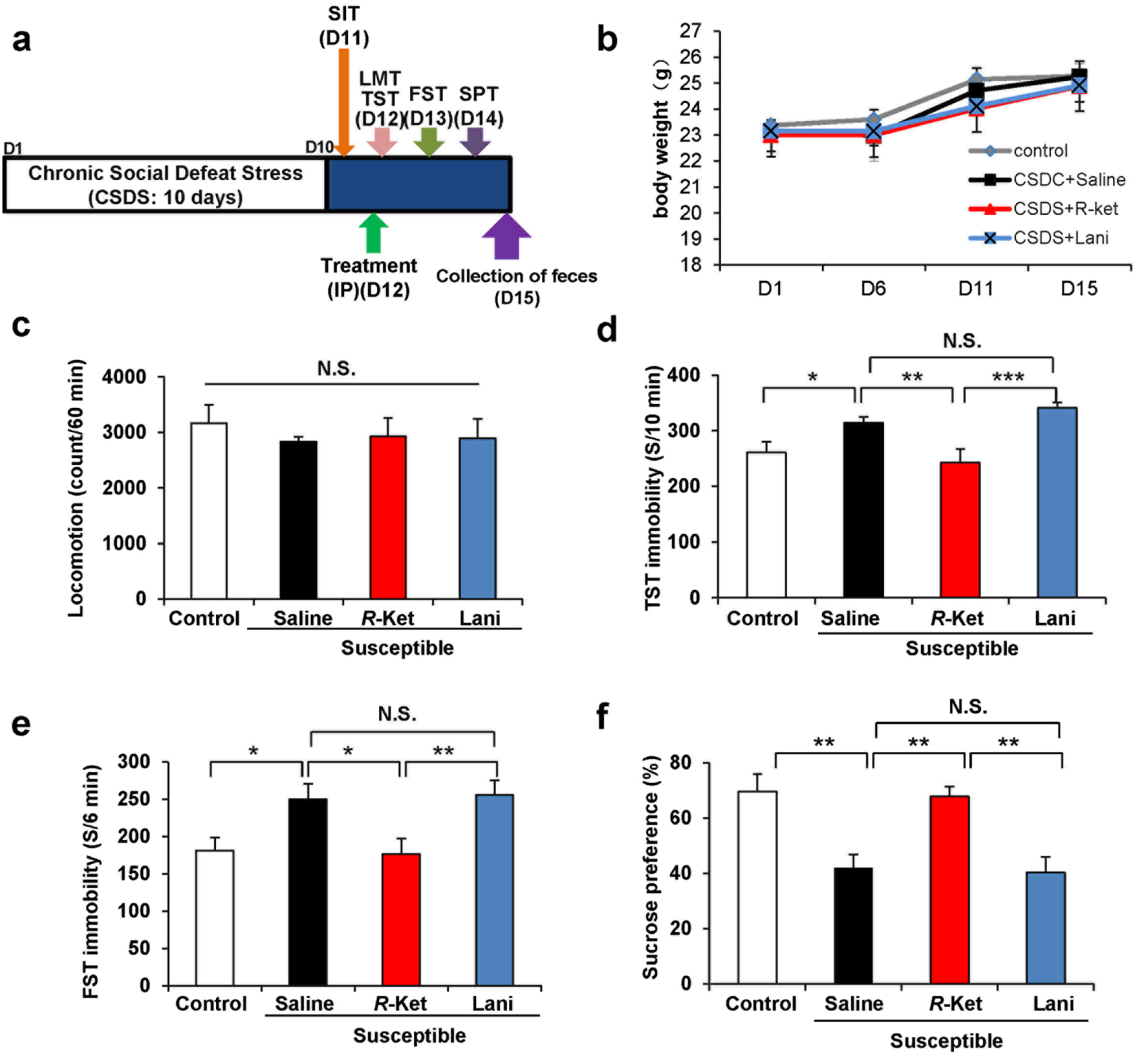
Effects of (*R*)-ketamine and lanicemine in the depression-like phenotype susceptible mice after CSDS. (**a**) The schedule of CSDS model, treatment, behavioral tests and feces collection. CSDS was performed from day 1 to day 10, and social interaction test (SIT) was performed on day 11. Saline (10 ml/kg), (*R*)-ketamine (10 mg/kg), or lanicemine (10 mg/kg) were administered i.p. into CSDS susceptible mice on day 12. Behavioral tests and SPT were performed form day 12 to day 14. On day 15, feces were collected. (**b**) Body weight (two-way ANOVA, time: F_3,15_ = 20.99, P < 0.001, treatment: F_3,15_ = 1.688, P = 0.176, interaction: F_9,15_ = 0.337, P = 0.960). (**c**–**e**) Behavioral tests including LMT (one-way ANOVA, F_3,20_ = 0.241, P = 0.867), TST (F_3,20_ = 7.025, P = 0.002) and FST (F_3,20_ = 4.722, P = 0.012) were performed. (**f**) SPT was performed 2 days after a single dose (F_3,20_ = 9.555, P < 0.001). Data are shown as mean ± S.E.M. (n = 6). *P < 0.05, **P < 0.01, ***P < 0.001. NS: not significant.

The social interaction test (SIT) after CSDS was performed on day 11 (Fig. 1a). In the SIT (no target), the social interaction time of two groups was the same (Fig. S1). In the SIT (target), we divided the susceptible or resilient mice by the evaluation of the time of mice in the interaction area (Fig. S1). The antidepressant effects of (*R*)-ketamine (10 mg/kg) and lanicemine (10 mg/kg) in susceptible mice after CSDS were examined (Fig. 1a). There were no changes in the body weight among the four groups (Fig. 1b). There were also no significant differences in the locomotion among the four groups (Fig. 1c). In the tail suspension test (TST) and forced swimming test (FST), (*R*)-ketamine, but not lanicemine, significantly decreased the increased immobility time in the susceptible mice (Fig. 1d and e). In the sucrose preference test (SPT), (*R*)-ketamine exerted potent anti-anhedonia effects two days after a single dose (Fig. 1f). These data indicate that (*R*)-ketamine exerts potent antidepressant and anti-anhedonia effects in a CSDS model, consistent with our previous reports^26,28,29^. In contrast, lanicemine did not show antidepressant effects in the CSDS model, consistent with the recent clinical results^34^.

### The Principal Coordinate Analysis (PCoA) of the gut bacterium data

**Figure 2:**
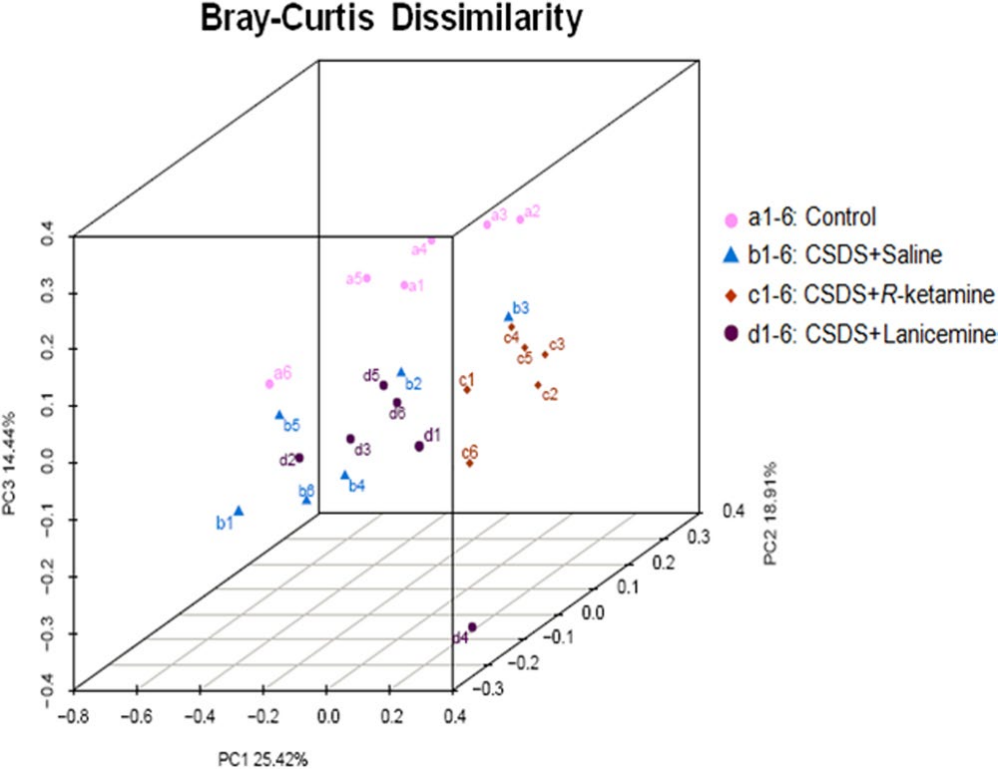
The PCoA of the gut bacterium data. The PCoA analysis plots of Bray-Curtis dissimilarity among the four groups (n = 6).

The PCoA analysis plots of Bray-Curtis dissimilarity among the four groups showed that the dots of (*R*)-ketamine treated group (c1-c6) were different from the dots of saline treated group (b1-b6) whereas the dots of lanicemine treated group (d1-d6) were similar to the dots of saline treated group (b1-b6) (Fig. 2a). Thus, it is likely that (*R*)-ketamine has more potency to improve the altered composition of gut microbiota after CSDS than lanicemine.

### Composition in the gut bacteria at the levels of phylum and class

**Figure 3:**
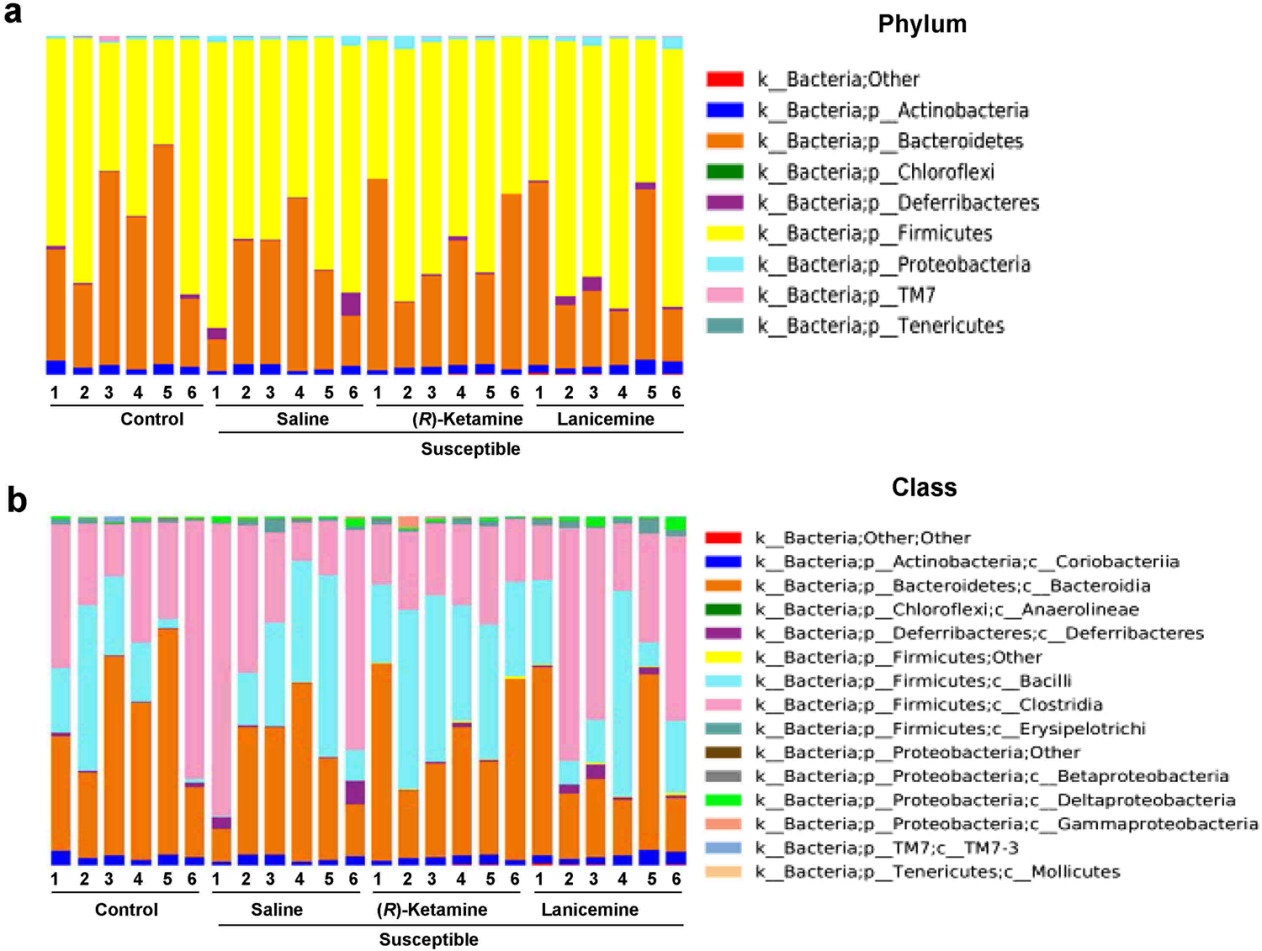
Composition in the gut bacterium at the levels of phylum and class. (**a**) The relative abundances of phylum in fecal samples of the four groups 3 days after a single dose of saline, (*R*)-ketamine or lanicemine. The relative abundances of *Bacteroidetes* and *Firmicutes* were shown in the all groups. (**b**) The relative abundances of class in fecal samples of the four groups 3 days after a single dose of saline, (*R*)-ketamine or lanicemine. The relative abundances of *Bacteroidia*, *Bacilli*, and *Clostridia* were shown in the all groups. Data of each mouse are shown (n = 6).

The compositions of gut bacterium of feces from all groups 3 days after a single dose of saline, (*R*)-ketamine or lanicemine were determined using 16S ribosomal RNA gene sequencing. At the level of phylum, the relative abundances of *Bacteroidetes* and *Firmicutes* were shown in the all groups (Fig. 3a). At the level of class, the relative abundances of *Bacteroidia*, *Bacilli*, and *Clostridia* were shown in all groups (Fig. 3b). At both levels, there were no significant differences among the four groups (Fig. 3a and b).

### Altered composition in the gut bacteria at the order level

**Figure 4:**
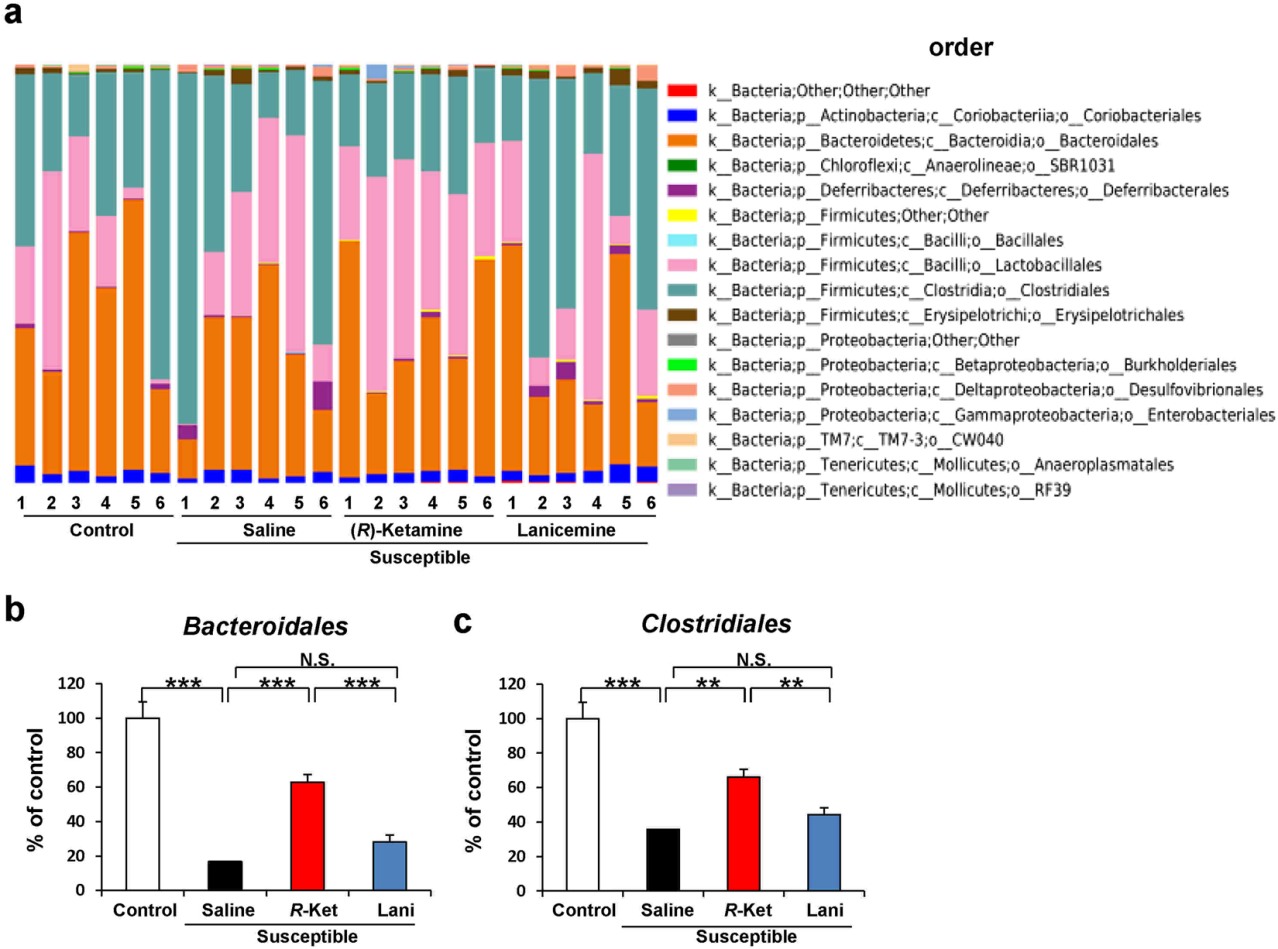
Altered composition in the gut bacteria at the order level. (**a**) The relative abundances of order in fecal samples of the four groups 3 days after a single dose of saline, (*R*)-ketamine or lanicemine. (**b**)The order levels of *Bacteroidales* were significantly altered (one-way ANOVA: F_3,20_ = 45.166, P < 0.001). (**c**) The order level of *Clostridiales* were significantly altered (one-way ANOVA: F_3,20_ = 30.221, P < 0.001). Data are shown as mean ± S.E.M. (n = 6). **P < 0.01, ***P < 0.001. NS: not significant.

The order level of gut bacterium 3 days after a single dose of saline, (*R*)-ketamine or lanicemine are shown (Fig. 4a). At the order level, *Bacteroidales* and *Clostridiales* were significantly lower in the CSDS susceptible mice than in the control mice. Furthermore, (*R*)-ketamine significantly improved the reduced levels of *Bacteroidales* and *Clostridiales* in the CSDS susceptible mice (Fig. 4b and c). In contrast, lanicemine did not alter the levels of these two bacteria (Fig. 4b and c).

### Altered composition in the gut bacteria at the family level

**Figure 5:**
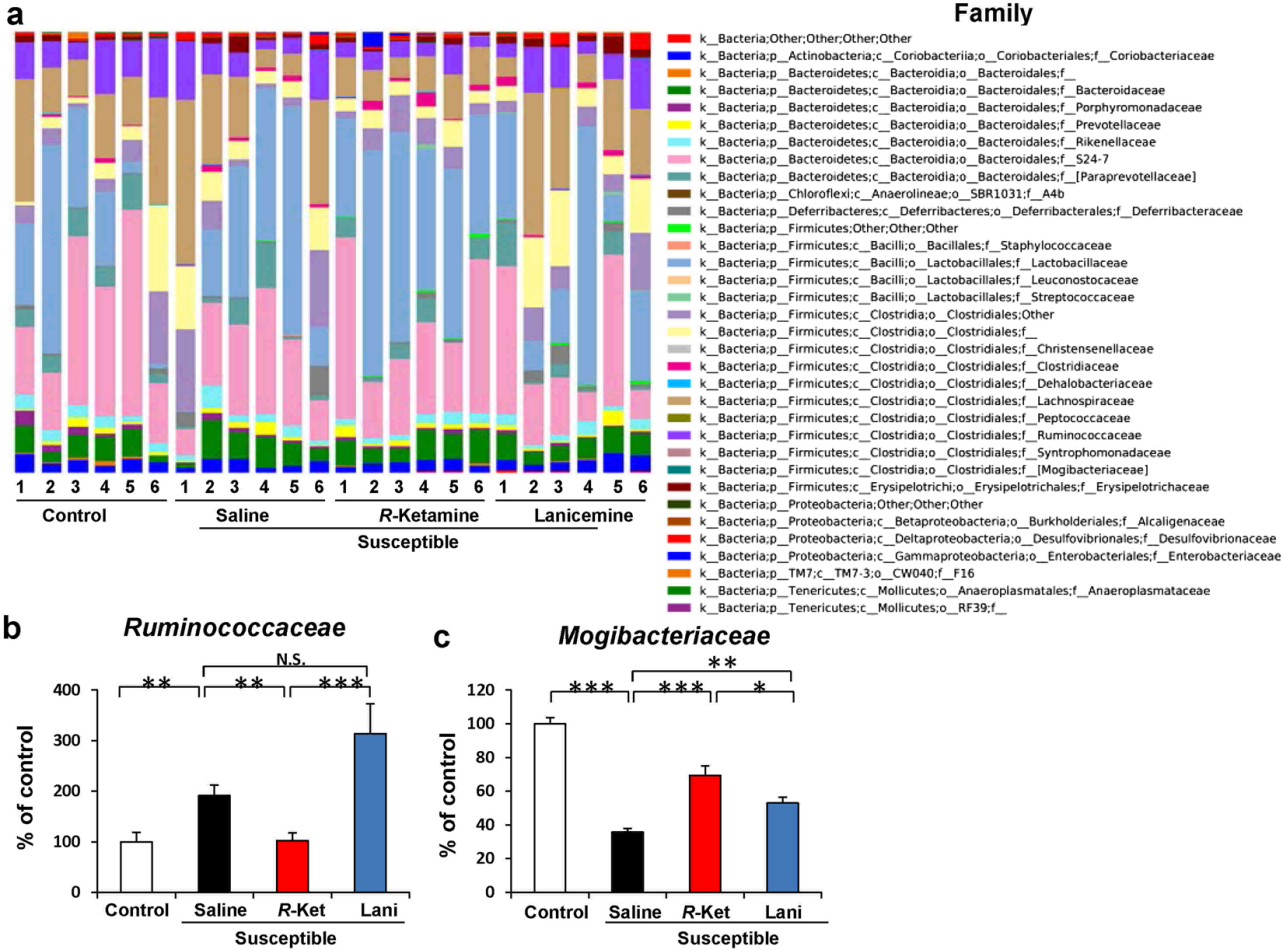
Altered composition in the gut bacteria at the family level. (**a**) The relative abundances of family in fecal samples of the four groups 3 days after a single dose of saline, (*R*)-ketamine or lanicemine. (**b**) The family levels of *Ruminococaceae* were significantly altered (one-way ANOVA: F_3,20_ = 32.341, P < 0.001). (**c**) The family levels of *Mogibacteriaceae* were significantly altered (one-way ANOVA: F_3,20_ = 49.097, P < 0.001). Data are shown as mean ± S.E.M. (n = 6). *P < 0.05, **P < 0.01, ***P < 0.001. NS: not significant.

The family levels of gut bacterium 3 days after a single dose of saline, (*R*)-ketamine or lanicemine are shown (Fig. 5a). At the family level, levels of *Ruminococcaceae* were significantly increased in the susceptible mice after CSDS. Furthermore, (*R*)-ketamine, but not lanicemine, significantly attenuated the increased levels of *Ruminococcaceae* in the susceptible mice (Fig. 5b). In contrast, levels of *Mogibacteriaceae* were significantly decreased in the susceptible mice. Furthermore, both (*R*)-ketamine and lanicemine significantly attenuated the reduced levels of *Mogibacteriaceae* in the susceptible mice. Notably, (*R*)-ketamine was more potent than lanicemine at increasing the levels of *Mogibacteriaceae* in the susceptible mice (Fig. 5c).

### Altered composition in the gut bacteria at the genus level

**Figure 6:**
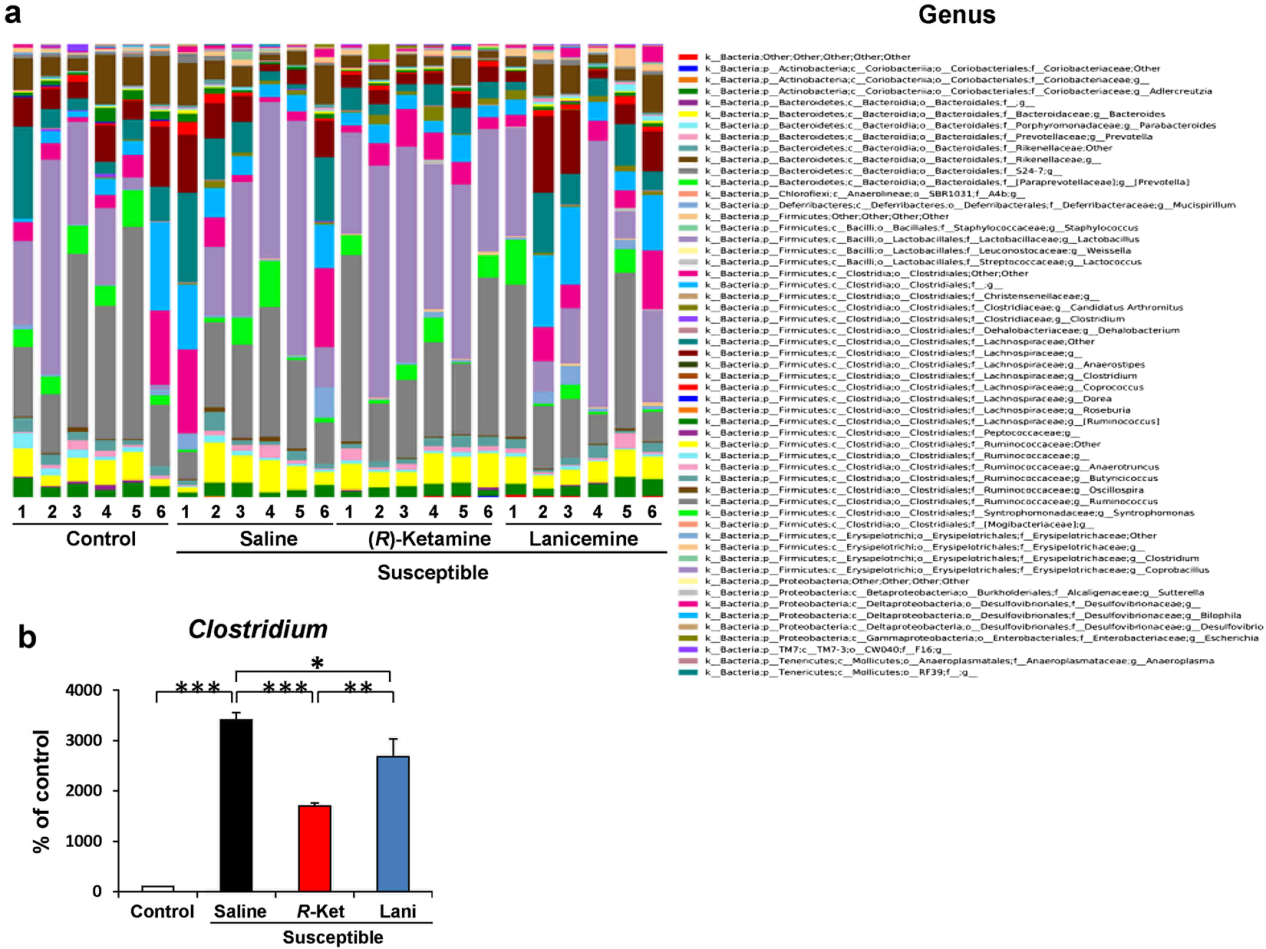
Altered composition in the gut bacteria at the genus level. (**a**) The relative abundances of genus in fecal samples of the four groups 3 days after a single dose of saline, (*R*)-ketamine or lanicemine. (**b**) The genus levels of *Clostridium* were significantly altered (one-way ANOVA: F_3,20_ = 55.538, P < 0.001). Data are shown as mean ± S.E.M. (n = 6). *P < 0.05, **P < 0.01, ***P < 0.001.

The genus levels of gut bacterium 3 days after a single dose of saline, (*R*)-ketamine or lanicemine are shown (Fig. 6a). At the genus level, levels of *Clostridium* were dramatically increased in the susceptible mice after CSDS. Furthermore, both (*R*)-ketamine and lanicemine significantly attenuated the increased levels of *Clostridium* in the susceptible mice. Notably, (*R*)-ketamine was more potent than lanicemine in reducing the levels of *Clostridium* in the susceptible mice (Fig. 6b).

## Discussion

The major findings of the present study are as follows: First, (*R*)-ketamine showed rapid and sustained antidepressant effects in a CSDS model of depression. In contrast, lanicemine did not show antidepressant effects in the same model. A previous study showed that inhibitory effects on *in vivo* [^3^H]MK-801 binding in the mouse brain by ketamine (10 mg/kg) and lanicemine (10 mg/kg) were the same^35^. It is, therefore, unlikely that NMDAR inhibition may play a role in the differential effects of (*R*)-ketamine and lanicemine although a further study is needed. Second, at the level of order, *Bacteroidales and Clostridiales* were significantly decreased in the CSDS susceptible mice. Interestingly, (*R*)-ketamine, but not lanicemine, significantly attenuated the reduced levels of *Bacteroidales and Clostridiales* in the susceptible mice. Third, at the family level, *Ruminococcaceae* and *Mogibacteriaceae* were significantly altered in the CSDS susceptible mice. Interestingly, (*R*)-ketamine, but not lanicemine, significantly attenuated the increased levels of *Ruminococcaceae* in the susceptible mice. Furthermore, both (*R*)-ketamine and lanicemine significantly attenuated the reduced levels of *Mogibacteriaceae* in the susceptible mice although the effects of lanicemine were less potent than (*R*)-ketamine. Finally, (*R*)-ketamine significantly attenuated the increased levels of *Clostridium*, the genus of bacteria, in the susceptible mice although the effects of lanicemine were less potent than (*R*)-ketamine. These findings suggest that altered composition of gut microbiota in the CSDS susceptible mice might play a role in the depression-like phenotype, and that the improvement of the altered composition of gut microbiota in the susceptible mice by (*R*)-ketamine might play, in part, a role in its rapid antidepressant effect.

At the order level, we found marked reductions of *Bacteroidales* and *Clostridiales* levels in the susceptible mice after CSDS. There was a report showing altered levels of *Bacteroidales* in patients with depression^46^. In addition, low levels of *Bacteroidales* are associated with obesity; which is related with depression through inflammation^47,48^. There was a significant increase in *Bacteroidales* in high-fat fed rats compared with low-fat fed rats^49^. Furthermore, levels of *Bacteroidales* were correlated significantly to better memory performance in the mice on a high-fat diet^50^, suggesting a possible link between food-related behaviors and gut microbiota. Collectively, it seems that reduced levels of *Bacteroidales* might play a role in the pathogenesis of depression.

The order *Clostridiales* include the polysaccharolytic and gram-positive bacteria that contribute strongly to the production of short chain fatty acids (e.g., butyrate) in the gut^51^. A randomized, double-blind, placebo-controlled study showed that probiotic intake markedly redistributed the microbial taxa of *Clostridiales* ^52^. In this study, we found that (*R*)-ketamine, but not lanicemine, significantly improved the reduced levels of *Bacteroidales* and *Clostridiales* in a CSDS model, consistent with the beneficial antidepressant effects of (*R*)-ketamine. Therefore, it is likely that the restoration of *Bacteroidales* and *Clostridiales* by (*R*)-ketamine may partly explain its robust antidepressant actions. Nonetheless, further studies underlying the role of *Bacteroidales* and *Clostridiales* in the antidepressant actions of (*R*)-ketamine are needed.

*Ruminococcaceae* is the family of the class *Clostridia* which are anaerobic, Gram-positive microbes. *Ruminococcaceae* is also known to be cellulolytic, as well as active in acetate, formate, and hydrogen production^53^. In healthy Japanese adults, the scores for bowel movement frequency were significantly correlated with the abundances of *Mogibacteriaceae* in the fecal microbiota^54^. Although the exact physiological functions of *Ruminococcaceae* and *Mogibacteriaceae* are not fully understood, it is likely that the altered composition of these microbiota in the gut may contribute to the pathogenesis of depression. Interestingly, we found that (*R*)-ketamine, but not lanicemine, significantly attenuated the increased levels of *Ruminococcaceae* in CSDS susceptible mice. We also found that both (*R*)-ketamine and lanicemine significantly attenuated the reduced levels of *Mogibacteriaceae* in CSDS susceptible mice. Interestingly, the increase in the levels of *Mogibacteriaceae* induced by (*R*)-ketamine was more potent than that by lanicemine. Although the precise physiological implications of *Ruminococcaceae* and *Mogibacteriaceae* in depression are unknown, it is likely that the restoration in the level of these bacteria induced by (*R*)-ketamine partially mediates its antidepressant action. Nonetheless, further studies on the relationship between (*R*)-ketamine’s antidepressant effects and these bacteria are needed.

*Clostridium* is a genus of Gram-positive bacteria, which includes several significant human pathogens, including the causative agent of botulism and an important cause of diarrhea *Clostridium difficile*. A study showed that adults with depression seem to be more likely to develop *Clostridium difficile* infection^55^. In addition, patients with depressive symptoms exhibit greater and more prolonged inflammatory responses after antigen challenge than subjects without depressive symptoms, suggesting that depression may result in immune dysregulation^56^. A recent study reported increased levels of *Clostridium* in actively-depressed patients compared to healthy subjects^37^. Furthermore, there was a negative correlation between *Clostridium* levels and serum levels of brain-derived neurotrophic factor in depressed patients^37^. In this study, we found marked increases of *Clostridium* in CSDS susceptible mice. We also found that (*R*)-ketamine significantly attenuated the increased levels of *Clostridium* in the susceptible mice. Interestingly, a decrease in the levels of *Clostridium* induced by (*R*)-ketamine was more potent than that by lanicemine. Taken together, it is likely that the restoration of *Clostridium* by (*R*)-ketamine may partly explain its robust antidepressant actions. Nonetheless, further studies on the role of the order *Clostridiales*, including the genus *Clostridium*, in depression and antidepressant actions of (*R*)-ketamine are needed.

The present data do not provide direct evidence of the effect of gut microbiota on the antidepressant actions of (*R*)-ketamine because behavioral experiments using germ-free mice were not performed. However, it is well known that the anti-microbial effects of currently available antidepressants are important for the correction of the intestinal dysbiosis observed in depressed patients^45^. Therefore, the gut microbiota–brain axis possibly plays a role in the antidepressant actions of (*R*)-ketamine. Nonetheless, additional studies elucidating the relationship between the gut microbiota axis and the antidepressant actions of (*R*)-ketamine are needed.

In conclusion, the present study suggests that the gut microbiota–brain axis might be associated with the antidepressant actions of (*R*)-ketamine, namely via (*R*)-ketamine-induced changes in the levels of the order *Bacteroidales*, *Clostridiales;* the family *Ruminococcaceae*, *Mogibacteriaceae*; and the genus *Clostridium*.

## Methods and Materials

### Animals

Male adult C57BL/6 mice, aged 8 weeks (body weight 20–25 g, Japan SLC, Inc., Hamamatsu, Japan) and male adult CD1 (ICR) mice, aged 13–15 weeks (body weight > 40 g, Japan SLC, Inc., Hamamatsu, Japan) were used. Animals were housed under controlled temperatures and 12 hour light/dark cycles (lights on between 07:00–19:00 h), with ad libitum food (CE-2; CLEA Japan, Inc., Tokyo, Japan) and water. The protocol was approved by the Chiba University Institutional Animal Care and Use Committee. This study was carried out in strict accordance with the recommendations in the Guide for the Care and Use of Laboratory Animals of the National Institutes of Health, USA.

### Materials

(*R*)-ketamine hydrochloride was prepared by recrystallization of (*R*,*S*)-ketamine (Ketalar^®^, ketamine hydrochloride, Daiichi Sankyo Pharmaceutical Ltd., Tokyo, Japan) and D-(−)-tartaric acid, as described previously^25^. The purity of (*R*)-ketamine was determined by a high-performance liquid chromatography (CHIRALPAK^®^ IA, Column size: 250 × 4.6 mm, Mobile phase: n-hexane/dichloromethane/diethylamine (75/25/0.1), Daicel Corporation, Tokyo, Japan). The dose (10 mg/kg as ketamine hydrochloride) of (*R*)-ketamine and lanicemine (AZD6765; Sigma-Aldrich Co. Ltd, St Louis, MO, USA) was used as previously reported^26–30,57^.

### Chronic social defeat stress (CSDS) model

The procedure of CSDS was performed as previously reported^26,28,29,57–59^. Every day the C57BL/6 mice were exposed to a different CD1 aggressor mouse for 10 min, total for 10 days. When the social defeat session ended, the resident CD1 mouse and the intruder mouse were housed in one half of the cage separated by a perforated Plexiglas divider to allow visual, olfactory, and auditory contact for the remainder of the 24-h period. At 24 h after the last session, all mice were housed individually. On day 11, a social interaction test (SIT) was performed to identify subgroups of mice that were susceptible and unsusceptible (resilient) to social defeat stress. This was accomplished by placing mice in an interaction test box (42 × 42 cm) with an empty wire-mesh cage (10 × 4.5 cm) located at one end. The movement of the mice was tracked for 2.5 min, followed by 2.5 min in the presence of an unfamiliar aggressor confined in the wire-mesh cage. The duration of the subject’s presence in the “interaction zone” (defined as the 8-cm-wide area surrounding the wiremesh cage) was recorded by a stopwatch. The interaction ratio was calculated as time spent in an interaction zone with an aggressor/time spent in an interaction zone without an aggressor. An interaction ratio of 1 was set as the cutoff: mice with scores <1 were defined as “susceptible” to social defeat stress and those with scores ≥1 were defined as “unsusceptible”. Approximately 70–80% of mice were susceptible after CSDS (Fig. S1). Susceptible mice were randomly divided in the subsequent experiments. Control mice without social defeat stress were housed in the same cage before the behavioral tests.

### Treatment and behavioral tests

Saline (10 ml/kg), (*R*)-ketamine (10 mg/kg) or lanicemine (10 mg/kg) was administered intraperitoneally (i.p.) into the susceptible mice after CSDS. Saline (10 ml/kg) was also administered i.p. into control mice (Fig. 1a). Behavioral tests, including locomotion test (LMT), tail suspension test (TST), forced swimming test (FST) and 1% sucrose preference test (SPT), were performed as reported previously^26,28,29,57–59^. Behavioral test were also performed by two observers who were blinded to the group assignment of mice. Each treatment group was equally represented in each experimental cohort.

#### Locomotion

The locomotor activity was measured by an animal movement analysis system SCANETMV-40 (MELQUEST Co., Ltd., Toyama, Japan). The mice were placed in experimental cages (length × width × height: 560 × 560 × 330 mm). The cumulative exercise was recorded for 60 minutes. Cages were cleaned between testing session.

#### TST

A small piece of adhesive tape placed approximately 2 cm from the tip of the tail for mouse. A single hole was punched in the tape and mice were hung individually, on a hook. The immobility time was recorded for 10 minutes. Mice were considered immobile only when they hung passively and completely motionless.

#### FST

The FST was tested by an automated forced-swim apparatus SCANETMV-40 (MELQUEST Co., Ltd., Toyama, Japan). The mice were placed individually in a cylinder (diameter: 23 cm; height: 31 cm) containing 15 cm of water, maintained at 23 ± 1 °C. Immobility time from activity time as (total) – (active) time was calculated by the apparatus analysis software. The immobility time for mouse was recorded for 6 minutes.

#### SPT

Mice were exposed to water and 1% sucrose solution for 48 h, followed by 4 hours of water and food deprivation and 1 hour exposure to two identical bottles, one is water, and another is 1% sucrose solution. The bottles containing water and sucrose were weighed before and at the end of this period. The sucrose preference was calculated as a percentage of sucrose solution consumption to the total liquid consumption.

### 16S rRNA analysis of fecal samples

The fecal samples were collected 3 days after a single dose of saline (10 ml/kg), (*R*)-ketamine (10 mg/kg) or lanicemine (10 mg/kg). They placed in 1.5 ml tubes, snap-frozen on dry ice and stored at −80 °C. The 16S rRNA analysis of fecal samples was performed at Takara Bio. Inc. (Shiga, Japan). The DNA extraction was performed using the MoBio Powerlyzer Powersoil DNA Isolation Kit (MoBio Laboratories, Carlsbad, CA, USA). The V4 hypervariable region of the bacterial 16S rRNA gene was amplified from the fecal DNA extracts using modified universal bacterial primer pairs 515 F (5′-TCGTCGGCAGCGTCAGATGTGTATAAGAGACAGGTGCCAGCMGCCGCGGTAA-3′) and 806 R (5′-GTCTCGTGGGCTCGGAGATGTGTATAAGAGACAGGGACTACHVGGGTWTCTAAT-3′) with Illumina adaptor overhang sequences. Amplicons were generated, cleaned, indexed and sequenced according to the Illumina MiSeq 16S Metagenomic Sequencing Library Preparation protocol (http://support.illumina.com/ downloads/16s_metagenomic_sequencing_library_preparation.html) with certain modifications. Sequencing data were combined and sample identification assigned to multiplexed reads using the MOTHUR software environment^42,60,61^. The data were denoised; low quality sequences, pyrosequencing errors, and chimeras were removed, and then sequences were clustered into operational taxonomic units (OTUs) at 97% identity using the CD-HITOTU pipeline (available from http://eeizhong-lab.ucsd.edu/cd-hit-otu)^43,61,62^. OTUs containing fewer than four reads per individual diet/animal combination were excluded due to the likelihood of there being a sequencing artifact. The samples were normalized by randomly resampling sequences used to the lowest number of sequences per sample (each diet/animal combination) using Daisychopper (http://www.festinalente.me/bioinf/). Taxonomic classification of OTUs was conducted using the Ribosomal Database Project Classifier^43,61,63^.

### Statistical analysis

The data show as the mean ± standard error of the mean (S.E.M.). Analysis was performed using PASW Statistics 20 (formerly SPSS Statistics; SPSS, Tokyo, Japan). The data were analyzed using the one-way analysis of variance (ANOVA) or two-way ANOVA, followed by *post-hoc* Tukey test. Furthermore, Principal Coordinate Analysis (PCoA) was performed to visualize similarities or dissimilarities of the data of four groups. The P-values of less than 0.05 were considered statistically significant.

## Electronic supplementary material

Supplemental information
