# Supplementary material for: Comparison of (R)-ketamine and lanicemine on depression-like phenotype and abnormal composition of gut microbiota in a social defeat stress model
Source: Sci Rep. 2017 Nov 16;7:15725. doi: 10.1038/s41598-017-16060-7 (PMC5691133; doi:10.1038/s41598-017-16060-7)
Supplement: Supplementary file 1 — Supplemental information [file 41598_2017_16060_MOESM1_ESM.pdf]

Supplemental information

Comparison of (*R*)-ketamine and lanicemine on depression-like phenotype and abnormal composition of gut microbiota in a social defeat stress model

Youge Qu, Chun Yang, Qian Ren, Min Ma, Chao Dong, Kenji Hashimoto

Division of Clinical Neuroscience, Chiba University Center for Forensic Mental Health, Chiba, Japan

Correspondence: Dr. Kenji Hashimoto, Division of Clinical Neuroscience, Chiba University Center for Forensic Mental Health, Chiba 260-8670, Japan
Tel: +81-43-226-2517; Fax: +81-43-226-2561 (e-mail: hashimoto@faculty.chiba-u.jp)

Current position of Dr. Chun Yang: Department of Anesthesiology, Tongji Hospital, Tongji Medical College, Huazhong University of Science and Technology, Wuhan 430030, China

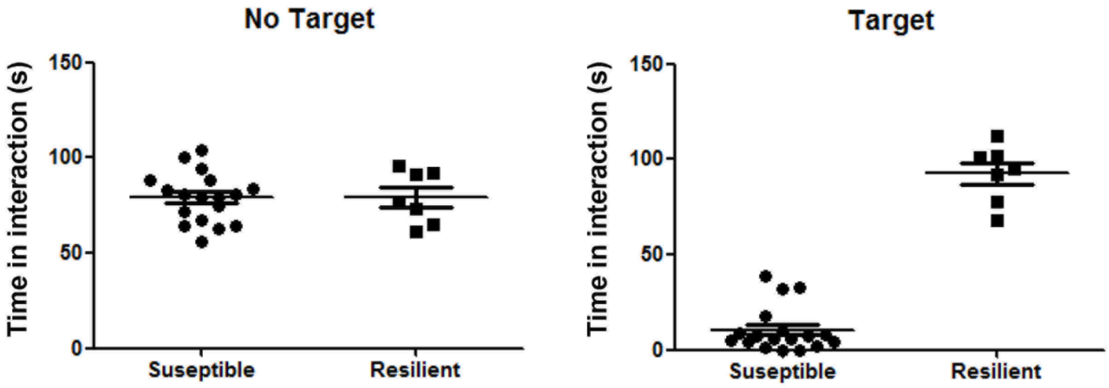

Figure S1. The social interaction test (SIT) after CSDS.
In the SIT (no target), there was no changes both groups. In the SIT (target), we divided the susceptible mice (n = 18) and resilient mice (n = 7) by the evaluation of the time of mice in the interaction area. Subsequently, the susceptible mice (n = 18) were randomly divided into three groups (saline, (*R*)-ketamine, lanicemine).
